# The long-read assembly of *Apareiodon* sp., a neotropical fish with a ZZ/ZW sex chromosome system

**DOI:** 10.1590/1678-4685-GMB-2024-0098

**Published:** 2024-10-11

**Authors:** Ivan Rodrigo Wolf, Michelle Orane Schemberger, Matheus Azambuja, Fernanda Souza de Oliveira, Viviane Nogaroto, Guilherme Targino Valente, Cesar Martins, Marcelo Ricardo Vicari

**Affiliations:** 1 Universidade Estadual Paulista, Instituto de Biociências de Botucatu, Departamento de Morfologia, Botucatu, SP, Brazil.; 2 Universidade Federal do Paraná, Centro Politécnico, Programa de Pós-Graduação em Genética, Curitiba, PR, Brazil.; 3 Fundação Oswaldo Cruz (FIOCRUZ), Instituto Carlos Chagas, Laboratório da Regulação da Expressão Gênica, Curitiba, PR Brazil.; 4 Universidade Estadual de Ponta Grossa, Departamento de Biologia Estrutural, Molecular e Genética , Ponta Grossa, PR, Brazil.

**Keywords:** Chromosomes, genome sequence, Parodontidae, Teleostei

## Abstract

Neotropical fishes emerge as an extremely diverse group of vertebrates where genomic strategies to evaluate structural and functional features are still beginning. Here, we present a second draft genome of *Apareiodon* sp. (2n=54, ZZ/ZW), adding PacBio technology whole genome sequencing, and assembling by combining two technologies (long and short reads). Using a detailed strategy for genome assembly with fish genomes of *Pygocentrus nattereri*, *Carassius auratus*, and *Astyanax mexicanus* as references, the final assembly of the *Apareiodon* sp. genome generated 93 scaffolds, an N50 of 37,200,078 bases, and a size estimate considering 28 scaffolds (26 autosomes+ZW) of ~945 Mb. In *Apareiodon* sp., this second genome draft confirmed that ~36% of the genome is composed of repetitive DNA. Furthermore, the new draft genome has improved genomic quality assessments, allowing the annotation of 36,290 genes and 15,683 proteins, which presented similarities to reference genomes. The second draft genome of *Apareiodon* sp. will be useful for research on integrative cytogenetic and genomic data. It will open perspectives for analyzing sex-determining genes in Neotropical fish with a ZZ/ZW sex chromosome system.

*Apareiodon* sp. (2n=54, ZZ/ZW) is a Neotropical fish species (Actinopterygii: Parodontidae) that inhabits the high Tibagi river basin and differs from the congener *Apareiodon ibitiensis* by ~2% of Kimura two-parameter (K2P) genetic distance (Vicari et al., 2006; Nascimento, 2015). Parodontidae representatives are recognized to have few diagnostic morphological traits, and most of the continuous characters overlapping (Pavanelli, 2003). Despite morphological traits being quite similar between *Apareiodon* sp. and *A. ibitiensis*, cytogenetics and genetics analyses attest *Apareiodon* sp. as an undescribed species in Parodontidae (Vicari *et al*., 2006; Nascimento, 2015; Azambuja et al., 2023, among others). The remarkable chromosomal feature in this fish family is the differentiation of a ZZ/ZW or ZZ/ZW_1_W_2_ heteromorphic sex chromosome system in some species (Schemberger et al., 2011; Santos et al., 2019; Azambuja *et al*., 2022) compared to species that lack heteromorphic sex chromosomes and others with a recognizable proto-sex chromosome pair (Schemberger *et al*., 2011; Azambuja *et al*., 2023). Integrated cytogenetic and omics data have been used to understand sex chromosome differentiation in *Apareiodon* sp. (Schemberger *et al*., 2019; Azambuja *et al*., 2022). For this, a first genomic draft of *Apareiodon* sp. using short reads showed a size ~ 1 Gb and was assembled with ~ 42× and ~ 47× coverage for males and females, respectively (Schemberger *et al*., 2019).

Comparing data of fluorescence *in situ* hybridization and estimation of transposable elements (TEs) insertion times it was demonstrated that *Helitron*, *Tc1-Mariner*, and *CMC EnSpm* DNA transposons accumulated repeated copies during W chromosome differentiation between 20 and 12 million years ago (Schemberger et al., 2019). DNA transposons and microsatellite expansions appeared to be the major players in the W chromosome differentiation of *Apareiodon* sp. (Schemberger *et al*., 2019). However, the high genomic scaffold fragmentation was a barrier to determine gene content in the sex chromosome-specific region. Therefore, here we sequenced the genome of a female *Apareiodon* sp. using long reads, aiming to provide a new genome assembly, whith a better genome scaffolding and gene annotation. 

The long reads were obtained from the DNA isolated from a single female muscle of *Apareiodon* sp. 2n=54, ZW (from Rio Verde, Paraná State, Brazil, −25°04′35″ and −50°04′03″; Voucher number 3447 deposited in the Limnology, Ichthyology and Aquaculture Research Center Museum - NUPELIA of State University of Maringá, Paraná State, Brazil) by Novagene Technology Inc (California, USA) with Pacific Biosciences (PacBio). The long reads library was constructed using a PacBio Sequel SMRTbell Library prep and QC kit (Pacific Biosciences, CA, USA). Sequencing was performed using the PacBio Sequel II SMRTcell sequencing (Pacific Biosciences). Moreover, raw short-read data were obtained for two independent DNA libraries (100-base paired-end reads and 150-base paired-end reads) for each sex, i.e., male and female of *Apareiodon* sp. (Schemberger et al., 2019). The Illumina data was analyzed with FastQC (https://www.bioinformatics.babraham.ac.uk/projects/fastqc/) and then filtered with Trimmomatic (Bolger et al., 2014) to remove low-quality reads (Q Score < 33) and keep only reads >80 or >140 according to each library read size.

Quality control of long reads adapters and reads filtering steps were performed with Canu (Koren et al., 2017, 2018; Nurk et al., 2020). Long read assembly was done with Canu (Koren *et al*., 2017, 2018; Nurk *et al*., 2020) with the following parameters: correctedErrorRate=0.035 utgOvlErrorRate=0.065 trimReadsCoverage=2 trimReadsOverlap=500 (Figure 1 a ). The haplotype genome was recovered by Redundans (Pryszcz and Gabaldón, 2016) (Figure 1 b ). The assembly correction and polishing were performed in three rounds using Pilon (Walker et al., 2014) with the use of female short read libraries, resolving single base sequencing calling errors and removing miss-assemblies (Figure 1 c ).

**Figure 1 -:**
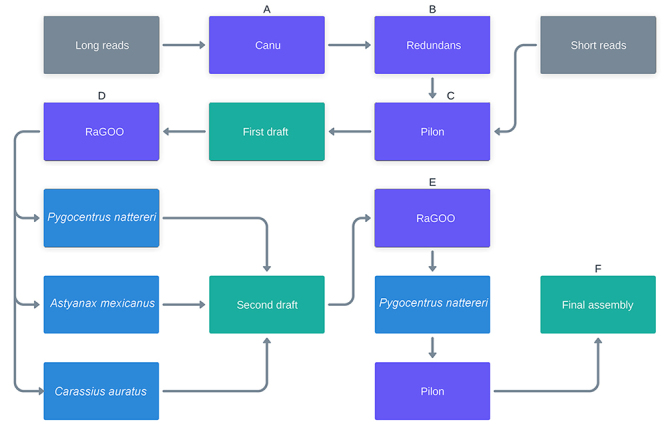
Assembly pipeline. Grey: Next-generation sequencing reads; Violet: Software; Blue: Reference genomes; Green: Assembly results.

Then, two scaffold procedures were made with RaGOO (Alonge et al., 2019) using the reference genomes of *Pygocentrus nattereri* (red-bellied piranha, NCBI RefSeq Access: GCF_015220715.1), *Carassius auratus* (goldfish, NCBI RefSeq Access: GCF_003368295.1), and *Astyanax mexicanus* (Mexican tetra, NCBI RefSeq Access: GCF_000372685.2) (Figure 1 d ). A second scaffolding procedure was made only with the *P. nattereri* reference genome, excluding *NC*_051239.1 and NC_051240.1 sequences (Figure 1 e ) followed by a final round of Pilon (Figure 1 f ).

Benchmarking Universal Single-Copy Orthologues (BUSCO) (Simão et al., 2015) were analyzed in gVolante (Nishimura et al., 2017) web interface with default parameters to assess the assembly completeness. Furthermore, QUAST was used to evaluate different versions of the assemblies (Gurevich et al., 2013), and GAScore was calculated as in Wolf et al. (2023), where the sum of squared differences in the assembly statistics is estimated to identify the sequence most similar to the reference considering 17 different features of QUAST (Table S1).

The genome annotation was performed with the BRAKER2 pipeline (Brůna et al., 2021), using the complete set of *P. nattereri*, *C. auratus* and *A. mexicanus* proteins as reference to perform the annotation. To obtain protein IDs to search against ontology databases easily, performed a protein Basic Local Alignment Search Tool - Blastp (Altschul et al., 1990) analysis of *Apareiodon* sp*.* annotated proteins and the *P. nattereri* protein database was performed. The IDs were filtered to keep the first ID with a similar protein length (>70%) and high sequence identity (>70%) for each annotation. Then, unused IDs and unidentified annotations were re-analyzed prioritizing sequence identity (>80%) and then protein length (>70%). The last redundancies were removed based on the identity criteria.

Finally, the TE was performed following the *de novo* annotation pipeline, according to Bell et al. (2022). TE search was performed with EDTA (Ou et al., 2019) and then classified into superfamilies and orders with DeepTE (Yan et al., 2020). The DeepTE library was used with RepeatMasker (Tarailo-Graovac and Chen, 2009) to identify and mask repeated sequences in the final assembly.

The long-read assembly followed by the short-read correction generated the first draft genome with 1,304 contigs (Table 1). The scaffolding based on three closely related species resulted in a second draft with 277 contigs (Table 1). In the next step, the scaffolding procedure with different references ranged from 92 to 240 scaffolds (Table 1), being *P. nattereri* the best reference.

**Table 1 -:** Assembly comparison and genome quality assessments.

Assembly	Number of Scaffolds/Contigs	N50	Size in 28th scaffolds	GAScore
Final scaffolding*	93	37,200,078	944,592,387	0.27
*Pygocentrus nattereri* ^*1*^	92	35,189,692	919,844,317	0.28
*Astyanax mexicanus* ^*2*^	151	34,741,361	894,735,468	0.29
*Carassius auratus* ^*3*^	240	28,077,891	843,352,896	0.30
Second draft	277	19,621,115	647,092,578	0.31
First draft	1304	2,115,861	198,642,493	0.41

The first draft is the assembly result from sequencing data. The second draft is the first draft scaffolding using the *Pygocentrus nattereri*, *Carassius auratus* and *Astyanax mexicanus* as references. ^1^ **:** All *Pygocentrus nattereri* chromosomes used as reference for scaffolding the second draft genome; ^2^ **:** All *Astyanax mexicanus* chromosomes used as a reference to scaffolding the second draft genome; ^3^ **:** All *Carassius auratus* chromosomes used as reference for scaffolding the second draft genome; *: Final assembly using the first 28 *Pygocentrus nattereri* chromosomes as a reference for scaffolding the second draft genome.

Since *Apareiodon* sp. contains 26 pairs of autosomes and one allosome pair, 2n=54, ZZ/ZW (Vicari et al., 2006), the genome size was checked considering the first 28 scaffolds (Table 1). Then, a final test was performed removing the two smallest chromosomes of *P. nattereri* (NC_051239.1 and NC_051240.1) prior to scaffolding. Thus, a smaller GAScore was obtained with a larger N50 and genome size (Table 1), being this draft considered the final assembly (Available at DDBJ/ENA/GenBank, under the accession JAWUEF01, GCA_040893165.1). The first genome assembly of *Apareiodon* sp. generated 660,652 scaffolds for males and 501,976 scaffolds for females (Schemberger et al., 2019). Therefore, the second draft genome presented here using long reads for a female version substantially improved the number of the contigs generated, N50, and other *Apareiodon* sp. genome quality assessments (Table S1). 

The BUSCO results showed more than 190 core genes out of 233, regardless of the analyzed assembly (Table 2), representing more than 80% of the expected core genes. Thus, genome quality assessments comparing orthologous genes (Waterhouse et al., 2017) showed that most of them were intact in the *Apareiodon* sp. genome. 

**Table 2 -:** BUSCO summarized results in the different versions of the genome assembly. About 80% of the core genes are found regardless of the assembly strategy.

	Missing	Partial	Complete
First draft	35 (15.02%)	5 (2.15%)	193 (82.83%)
Second draft	35 (15.02%)	3 (1.29%)	195 (83.69%)
Reference *A. mexicanus*	35 (15.02%)	4 (1.72%)	194 (83.26%)
Reference *C. auratus*	35 (15.02%)	2 (0.86%)	196 (84.12%)
Reference *P. nattereri*	35 (15.02%)	3 (1.29%)	195 (83.69%)
**Final Scaffolding**	**35 (15.02%)**	**3 (1.29%)**	**195 (83.69%)**

The annotation using BRAKER2 found 36,290 genes with 15,683 proteins similar to the reference genome. Furthermore, the gene annotation of more than 30 genes related to sex determination/differentiation opens perspective to analyze pathways in the gonadal differential of *Apareiodon* sp. In *Megaleporinus macrocephalus*, another Neotropical fish with a heteromorphic ZZ/ZW sex chromosome system, using dense omics strategies, the authors proposed highly differentiated regions in ZW chromosomes besides *Anti-Mullerian hormone receptor type II* (*Amhr2*) and *Bone morphogenetic protein* (*Bmp7*) as potential candidate genes for sex determination (Borges et al., 2024). However, the master sex-determining gene can vary in fish genomes, even in closely related species (Curzon et al., 2023). Thus, the genome of *Apareiodon* sp. could contribute to sex determination understanding and comparative genomic studies in Neotropical fishes.

Finally, the TE pipeline identified 6,057 element types amongst 37 known TE orders with 36.03% of the genome masked (Figure 2). The percentage of TEs in the genome was similar to that found by Schemberger et al. (2019), where the most abundant elements were grouped in the DNA transposon *Tc1-Mariner* superfamily. The homology-based method for TEs identification in *Apareiodon* sp. genome integrating cytogenetic data used by Schemberger *et al.* (2019) demonstrated *Helitron*, *Tc1-Mariner*, and *CMC EnSpm* elements in the differentiated W chromosome region. However, the fragmentation in the first genome draft impeded any conclusions regarding the genes in the specific sex-chromosome region. In the second genome draft presented here, mainly the scaffolds 4, 8, 12, and 25 were TE-enriched, opening possibilities for sex chromosome scaffold identification.

**Figure 2 -:**
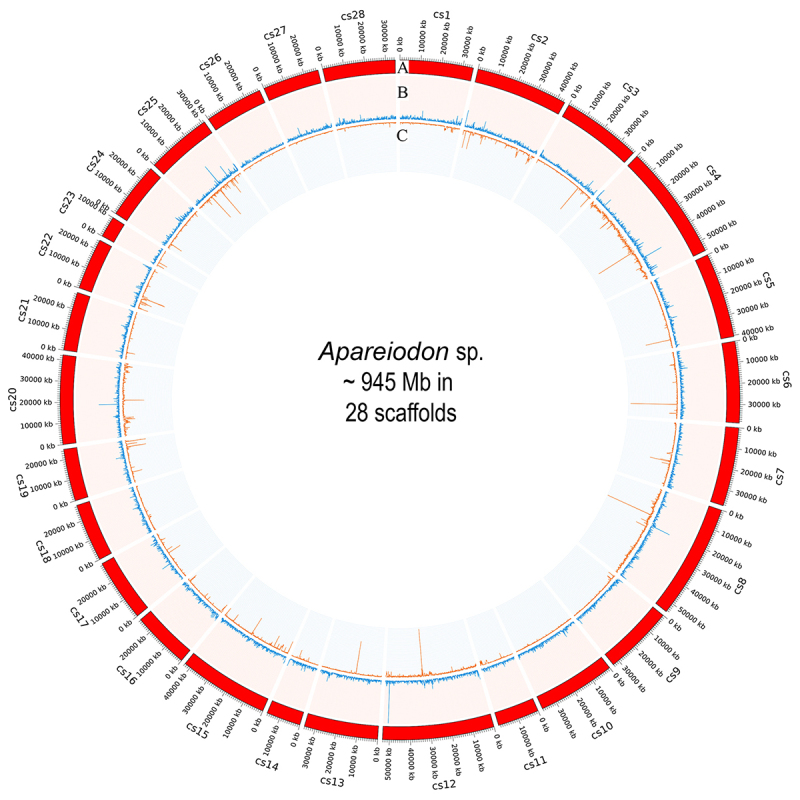
Genome overview in circos diagram. In (A) chromosome scaffold; (B) gene density; and (C) transposable element density. Densities were calculated in 50 kb intervals, scaffolds misaligned with the reference are not shown.

In summary, we have generated a good-quality long-read draft annotated genome for a Neotropical fish possessing a ZZ/ZW sex chromosome system. Since the role of repeat DNAs, mainly those related to transposable element functions, is poorly understood, this draft genome opens perspectives for studies on the organization, function of repeat DNAs, and chromosome evolution in Neotropical fishes. The genome assembly also opens the possibility of integrative cytogenetic and scaffold maps and recognition of genes in the sex determination cascade.

## Supplementary material

The following online material is available for this article:

Table S1 - Assembly statistics and *Apareiodon* sp. genome quality assessments.
