# Supplementary material for: The long-read assembly of Apareiodon sp., a neotropical fish with a ZZ/ZW sex chromosome system
Source: Genet Mol Biol. 2024 Oct 11;47(4):e20240098. doi: 10.1590/1678-4685-GMB-2024-0098 (PMC11468460; doi:10.1590/1678-4685-GMB-2024-0098)
Supplement: Table S1 - [file 1415-4757-GMB-47-4-e20240098-s1.pdf]

Genetics and Molecular Biology

DOI: https://doi.org/10.1590/1678-4685-GMB-2024-0098

Supplementary Material to "The long-read assembly of *Apareiodon* sp., a neotropical fish with a ZZ/ZW sex chromosome system"

Table S1 - Assembly statistics and *Apareiodon* sp. genome quality assessments.

Assembly	Contigs (≥ 0 bp)	Contigs (≥ 25000 bp)	Total length (≥ 1000 bp)	GC (%)	N50	N90	L90	Unaligned length	Genome fraction (%)	Duplication ratio	N's per 100 kbp	Total aligned length	Unaligned total	Unaligned partial	GAScore
Pygocentrus nattereri[Reference]	30	30	1,221,184,140	40.58	42,283,192	31,727,646	26	26,033	99.998	1	831,9	1,210,983,052	0	6	0
Final scaffolding*	93	89	954,416,576	39.92	37,200,078	24,923,301	24	946,485,806	0.336	1,731	93,43	7,042,186	29	64	0,26179384
Pygocentrus nattereri[1]	92	88	954,416,676	39.92	35,189,692	19,886,528	25	946,486,657	0.336	1,732	93,44	7,041,341	28	64	0,27402900
Astyanax mexicanus[2]	151	145	954,410,776	39.92	34,741,361	13,682,375	26	946,475,069	0.337	1,730	92,82	7,052,975	31	120	0,28938861
Carassius auratus[3]	240	234	954,401,876	39.92	28,077,891	10,212,165	30	946,462,094	0.337	1,730	91,89	7,065,987	39	201	0,31064369
Second draft	277	271	954,398,176	39.92	19,621,115	5,783,318	53	946,453,099	0.338	1,730	91,5	7,074,881	39	238	0,33423437
First draft	1,304	1,298	954,295,476	39.92	2,115,861	248,575	621	946,247,476	0.344	1,749	80,75	7,280,802	51	1253	0,45003459
